# The Trajectory of Children’s Social Competence from 18 Months to 30 Months of Age and Their Mother’s Attitude towards the Praise

**DOI:** 10.2188/jea.JE20090168

**Published:** 2010-03-05

**Authors:** Ryoji Shinohara, Yuka Sugisawa, Lian Tong, Emiko Tanaka, Taeko Watanabe, Yoko Onda, Yuri Kawashima, Maki Hirano, Etsuko Tomisaki, Yukiko Mochizuki, Kentaro Morita, Gan-Yadam Amarsanaa, Yuko Yato, Noriko Yamakawa, Tokie Anme, Japan Children’s Study Group

**Affiliations:** 1 Research Institute of Science and Technology for Society, Japan Science and Technology Agency, Tokyo, Japan; 2 Graduate School of Comprehensive Human Sciences, University of Tsukuba, Tsukuba, Ibaraki, Japan; 3 College of Letters, Ritsumeikan University, Kyoto, Japan; 4 Clinical Research Institute, Mie-Chuo Medical Center National Hospital Organization, Tsu, Japan

**Keywords:** child development, praise, social competence, caregiver-child interaction, longitudinal study

## Abstract

**Background:**

Praise from caregivers has been shown as an important influence on the development of social competence in early adolescence. However, the effects of praise in younger children have not been investigated. We examined how the trajectory of children’s social competence from 18 months to 30 months of age was related to their caregiver’s attitude towards the importance of praise at times when their child was 4 months and 9 months old.

**Methods:**

We studied 155 mother-child dyads, whose interactions during play were observed both when the child was 18 months and 30 months old, which was conducted as part of a Japan Science and Technology Agency (JST) project. The child’s social competence was assessed using the Interaction Rating Scale (IRS). Demographic data was obtained when the child was 4 months old, and the caregiver’s attitude towards the importance of praise when the child was both 4 months and 9 months old. A logistic regression analysis controlling for the effects of demographic variables was performed.

**Results:**

We found that children who had received continuous praise from their mother when they were 4–9 months of age had a decreased risk of low social competence at 18–30 months of age.

**Conclusions:**

A mother’s attitude towards the importance of praise at early stages of her child’s development has an important influence on the later trajectory of social competence.

## INTRODUCTION

Many studies have found that positive parenting behavior facilitates the development of children’s social competence, and that praise is one of the most important contributing factors.

Previous research into the effects of praise on children has shown that achievement incentive is influenced by the extent to which parents provide positive verbal feedback in the early stages of development,^1^^,^^2^ and that a high frequency of praise is associated with the development of high self-esteem.^2^^–^^4^ However, the subjects of these reports were 14-year-old children who had been studied from the age of two years, and there have been few studies on toddlers less than two years old.

An increase in antisocial behavior and aggression among school-aged children has led to an increased interest in the development of children’s social competence. However, in Japan the methods available for assessing children’s social competence are limited, and there is thus an urgent need to develop more suitable methods. This is important not only for research purposes, but also for health care and welfare specialists, who may be able to beneficially apply such methods when administering child care. We previously addressed this issue by developing a Japanese version of the Interaction Rating Scale (IRS), an easy-to-use measure that validly and reliably assesses several aspects of children’s social competence.^5^

In the present study, we examined the relationship between the attitude of caregivers to giving praise when their child was in infancy and the trajectory of their child’s social competence from 18 months to 30 months of age. Demographic variables with the potential to influence this relationship were controlled for.

## METHODS

### Participants and overview

Data was obtained from 199 mother-child dyads enrolled in a longitudinal study of social development conducted as part of a project initiated by the Japan Science and Technology Agency (JST). The children and their parents were recruited from two Japanese cities (Mie and Osaka). Laboratory-based videotaped observations of all dyads were made twice, once each when the child was 18 months and 30 months old in 2007 and 2008. However, analyses were restricted to 155 dyads for which the mother had provided demographic data and completed questionnaire items concerning caregiver attitudes towards the importance of praise when their child was 4 months and 9 months old.

All mothers provided informed written consent before participating in the study, the protocol of which was approved by the ethics committee of the JST.

### Measures

Demographic data and attitudes towards the importance of praise were obtained from a questionnaire administered as part of the larger longitudinal study from which the participants were drawn. Demographic variables included the gender of the child and whether they had any siblings, family type (nuclear or extended), the age and occupation of both parents, and the annual family income. The item addressing the caregiver’s attitude towards the importance of praise was “Praising child”; it had four response options: (1) Not at all important, (2) Of minor importance, (3) Fairly important, and (4) Very important. This item was completed by both the mother and father of the child.

The IRS was used to assess children’s social competence and mother’s child rearing competence. This measure has 10 subscales, five addressing each of child-related and caregiver-related factors. The child-related subscales are (1) Autonomy: Child initiates interaction with caregiver; (2) Responsiveness: Child is responsive to caregiver’s behavioral cues; (3) Empathy: Child behaves in accord with caregiver’s affective expression; (4) Motor regulation: Child’s behavior is clearly directed toward the task and he/she is not overactive/underactive; and (5) Emotional regulation: Child adjusts his/her emotional state to a comfortable level. The caregiver-related subscales are (1) Respect for the development of autonomy: Partner encourages child’s autonomy; (2) Respect for the development of responsiveness: Partner encourages child’s responsiveness; (3) Respect for the development of empathy: Partner encourages the child to develop empathy; (4) Respect for cognitive development: Caregiver encourages child’s cognitive development; and (5) Respect for social-emotional development: Caregiver encourages child’s social-emotional development. Scores for all IRS subscales were derived from laboratory-based observations of mother-child interactions.

### Mother-child interactions

Mother-child interactions took place in a playroom (4 m × 4 m) that was furnished with a small table and a child-sized chair, and that contained toys (small dolls, mini cars, and plastic toys). This was a relatively natural setting conducive to both spontaneous interaction and a certain degree of experimental control. The play activity was standardized in being restricted to building blocks, an age-appropriate activity popular in Japan, and began when the parent was given a box containing the blocks. This was followed by the child taking the blocks from the box, playing with them alongside their mother, and putting them back in the box. Mothers had been asked to play with their child as they normally would at home (providing instructions or help). The play activity lasted for 5–10 min. Three mother-child interactions were allowed in each task. Children who completed the task on the first attempt were not asked to perform any more tasks. Mother-child interactions were videotaped from five different angles (cameras were in each corner of the room and on the ceiling) and sound-recorded.

### Coding of mother-child interactions

It was necessary for coders to be sensitive to the cues of both mother and child, the latter of which could often seem ambiguous or confusing, and be transitory. Sensitivity was achieved via 1 month of training in the coding of videotaped parent-child interactions. Coders were doctoral or masters students who achieved at least 80% inter-rater agreement during training with pilot tapes. The reliability of the coding procedure was evaluated by having two coders each analyze a sample of tapes from the study (25% of the total), and was verified in their inter-rater agreement being high at 87%.

### Data treatment

#### Attitude variables

Responses to the “Praising child” item addressing a caregiver’s attitude towards the importance of praise were converted to a binary variable, with the response option “Very important” assigned a score of 1 (positive attitude) and all other response options assigned a score of 0 (negative attitude). From these values we constructed a variable representing the trajectory of the attitude towards praise across the times at which the child was 4 months and 9 months old. This variable had four categories: 0, reflecting a negative attitude at both ages (consistently negative); 1, reflecting a change in attitude from positive at 4 months to negative at 9 months (inconsistently negative); 2, reflecting a change in attitude from negative at 4 months to positive at 9 months (inconsistently positive); and 3, reflecting a positive attitude at both ages (consistently positive).

#### Children’s social competence

Child-related subscale items of the IRS were assigned a score of 1 if coded as “yes” and a score of 0 if coded as “no”; the summed score across all five subscales constituted the child’s total score (maximum = 25). For the ages of both 18 months and 30 months, children were grouped according to the quartile in which their total score fell: Those in the bottom quartile were assigned to a Low score group, and all others assigned to a High score group. Children in the Low score group at both 18 months and 30 months of age were further classified as belonging to a Low score transition group. The trajectory of children’s social competence from 18 months to 30 months of age was analyzed in terms of either belonging to the Low score transition group or not (all other children).

### Statistical approach

Chi-square tests were used to examine how the trajectory of children’s social competence from 18 months to 30 months of age related to each of the demographic variables and the trajectory of caregiver’s attitude towards the importance of praise.

A logistic regression analysis controlling for all demographic variables was performed to analyze the association between the trajectory of children’s social competence (dependent variable) and the trajectory of caregiver’s attitude towards praise (independent variable). The Statistical Analysis System (SAS) software package (Ver. 9.1) was used for all analyses, and *P* < 0.05 considered as statistically significant.

## RESULTS

For the ages of both 18 months and 30 months, the distribution of a High score group and a Low score group of children’s social competence are shown in Table 1. In terms of the trajectory of children’s social competence from 18 months to 30 months of age, 14 of the 155 children (9.1%) were classified as belonging to the Low score transition group (see Table 2). Table 3 shows the results of analyses concerning relationships between the trajectory of children’s social competence (Low score transition group vs. all others) and each of the demographic variables (the data for which were obtained when the child was 4 months old). The age of both the mother and father were the only variables significantly related to the trajectory of children’s social competence (*P* = 0.027 and *P* = 0.014, respectively).

**Table 1.** Group distribution of children’s social competence

Group	children’s social competence			

	18 months		30 months	
	
	*n*	%	*n*	%
Low score	45	29.0	28	18.1
High score	110	71.0	127	81.9

**Table 2.** Group distribution of the trajectory of children’s social competence

Group	*n*	%
The low score transition group	14	9.1
Other group	141	90.9

**Table 3.** Demographic information

Variable	Category	Trajectory of child’s social competence					

		Low score transition group		Others			
	
		*n*	%	*n*	%	*N*	%
Gender							
Boy	0	8	10.5	68	89.5	76	49.0
Girl	1	6	7.6	73	92.4	79	51.0
Family type							
Nuclear	0	12	9.0	121	91.0	133	75.6
Extended	1	2	9.1	20	90.9	22	12.5
Siblings							
No	0	10	11.8	75	88.2	85	54.8
Yes	1	4	5.7	66	94.3	70	45.2
Mother’s career							
No	0	3	4.6	62	95.4	65	41.9
Yes	1	11	12.2	79	87.8	90	58.1
Mother’s education							
Middle school	0	0	0.0	4	100.0	4	2.6
High school	1	3	7.1	39	92.9	42	27.1
Vocational school	2	2	5.9	32	94.1	34	21.9
Short-term college	3	3	8.8	31	91.2	34	21.9
University	4	6	15.4	33	84.6	39	25.2
Post college	5	0	0.0	2	100.0	2	1.3
Father’s career							
No	0	0	0.0	1	100.0	1	0.7
Yes	1	14	9.1	140	90.9	154	99.3
Father’s education							
Middle school	0	0	0.0	4	100.0	4	2.6
High school	1	4	6.3	59	93.7	63	40.6
Vocational school	2	1	6.3	15	93.8	16	10.3
Short-term college	3	1	25.0	3	75.0	4	2.6
University	4	6	11.1	48	88.9	54	34.8
Post college	5	2	14.3	12	85.7	14	9.0
Annual family income							
(Japanese yen)							
<2 million	0	1	14.3	6	85.7	7	4.5
2–4 million	1	2	4.5	42	95.5	44	28.4
4–6 million	2	6	8.3	66	91.7	72	46.5
6–8 million	3	4	20.0	16	80.0	20	12.9
8–10 million	4	1	16.7	5	83.3	6	3.9
≧10 million	5	0	0.0	6	100.0	6	3.9
Mother’s age (years)							
20–29	0	2	4.1	47	95.9	49	31.6
30–39	1	10	9.8	92	90.2	102	65.8
40–49	2	2	50.0	2	50.0	4	2.6
Father’s age (years)							
20–29	0	0	0.0	34	100.0	34	21.9
30–39	1	12	11.2	95	88.8	107	69.0
40–49	2	1	7.7	12	92.3	13	8.4
50–59	3	1	100.0	0	0.0	1	0.6

Table 4 shows data for caregiver’s attitude towards praise at each of 4 months and 9 months in relation to the trajectory of children’s social competence. Data and the results of analyses concerning the trajectory of caregiver’s attitude towards praise are shown in Table 5. There was a significant relationship between the trajectory of children’s social competence and the trajectory of the mother’s (*P* = 0.023), but not the father’s, attitude towards praise. These findings are supported by the results of our multiple logistic regression analysis, which revealed a significant (*P* = 0.015) and more than 3-fold increase in the risk of a child being in the Low transition group for every incremental step downwards in the mother’s trajectory of attitude towards praise score, from 3 (consistently positive) to 0 (consistently negative) (see Table 6).

**Table 4.** Caregiver’s attitude towards the importance of praise and their child’s later trajectory of social competence

Respondent	Child’s age (months)	Attitude	Binary attitude variable	The trajectory of child’s social competence					

				Low score transition group		Others			
	
				*n*	%	*n*	%	*N*	%
Mother	4	1. Not at all important	0	0	0.0	0	0.0	0	0.0
		2. Of minor importance		0	0.0	0	0.0	0	0.0
		3. Fairly important		1	20.0	4	80.0	5	3.2
		4. Very important	1	13	8.7	137	91.3	150	96.8

	9	1. Not at all important	0	0	0.0	0	0.0	0	0.0
		2. Of minor importance		0	0.0	1	100.0	1	0.6
		3. Fairly important		3	42.9	4	57.1	7	4.5
		4. Very important	1	11	7.5	136	92.5	147	94.8

Father	4	1. Not at all important	0	0	0.0	0	0.0	0	0.0
		2. Of minor importance		0	0.0	0	0.0	0	0.0
		3. Fairly important		0	0.0	8	100.0	8	5.2
		4. Very important	1	14	9.5	133	90.5	147	94.8

	9	1. Not at all important	0	0	0.0	0	0.0	0	0.0
		2. Of minor importance		0	0.0	1	100.0	1	0.6
		3. Fairly important		0	0.0	11	100.0	11	7.1
		4. Very important	1	14	9.8	129	90.2	143	92.3

**Table 5.** Caregiver’s trajectory of attitude towards the importance of praise and their child’s later trajectory of social competence

Respondent	Binary attitude variable		Trajectory of attitude	Trajectory of child’s social competence					

				Low score transition group		Others			
		
	4 months	9 months		*n*	%	*n*	%	*N*	%
Mother	0	0	0	1	100.0	0	0.0	1	0.6
	1	0	1	2	28.6	5	71.4	7	4.5
	0	1	2	0	0.0	4	100.0	4	2.6
	1	1	3	11	7.7	132	92.3	143	92.3

Father	0	0	0	0	4.4	3	10.1	3	1.7
	1	0	1	0	4.1	9	3.0	9	5.1
	0	1	2	0	7.2	5	4.5	5	4.0
	1	1	3	14	3.4	124	3.3	138	89.2

**Table 6.** Logistic regression analysis for the relationship between a mother’s trajectory of attitude towards the importance of praise and their child’s later trajectory of social competence

	Odds ratio	95% Cl
Trajectory of attitude		
praise	3.32^a^	1.26–8.74
Demographic variables controlled for		
Gender	1.62	0.43–6.15
Siblings	2.70	0.66–10.95
Family type	0.37	0.06–2.34
Mother’s career	0.35	0.08–1.62
Mother’s age	0.43	0.08–2.49
Father’s age	0.34	0.09–1.33
Family annual income	0.98	0.56–1.75
Mother’s education	0.77	0.45–1.33
Father’s education	0.75	0.47–1.22

^a^*P* < 0.05

## DISCUSSION

In the present study, we examined the longitudinal relationship between the trajectory of children’s social competence from 18 months to 30 months of age and the trajectory of caregiver’s attitude towards the importance of praise across the times at which the child was 4 months and 9 months old; potentially confounding demographic factors were controlled for in our analyses. We found that children whose mother had a consistently positive attitude towards praise had a decreased risk of low social competence at both 18 months and 30 months of age. These findings are very interesting in suggesting that a mother’s attitude towards the importance of praise when her child is as young as 4 months and 9 months old influences social competence at the later ages of 18 months and 30 months.

Many studies have shown positive parent-child interactions beyond infancy as promoting a high level of social competence in later stages of a child’s development. For mothers with a high level of social competence, both they and their children have been found to display more positive behaviors and emotions.^6^ It has also been reported that the preschool-aged children of mothers who allow autonomy in play and teaching tasks display more positive assertiveness.^7^ These relationships can be accounted for by such mothers responding to their child’s emotions often in constructive ways,^8^ and by the child imitating their mother’s social and emotional behavior patterns.^9^

We used a multiple logistic regression analysis model that adjusted for demographic variables potentially affecting the development of children’s social competence. It is considered that these variables affect the development of the child’s social competence, that is, birth order is related to the infants’ sociability toward peers,^10^ support (a family member) of caregivers is important for the development of a child,^11^ and the stressors (change in the occupation of parents, poverty, etc) in the home as the imminent environment of the child are related to children’s social difficulties.^12^^–^^14^

The results of the present study demonstrate a mother’s attitude towards the importance of praise at early stages of her child’s development (4 months and 9 months old) as an important influence on the trajectory of their social competence from 18 months to 30 months of age. Further research is needed to clarify the mechanisms by which this relationship operates.

## APPENDIX

### Japan children’s study group

Chairmen: Zentaro Yamagata (Department of Health Sciences, School of Medicine, University of Yamanashi), Hideaki Koizumi (Advanced Research Laboratory, Hitachi, Ltd.).

Participating Researchers: Kevin K. F. Wong, Yoko Anji, Hiraku Ishida, Mizue Iwasaki, Aya Kutsuki, Misa Kuroki, Haruka Koike, Daisuke N Saito, Akiko Sawada, Yuka Shiotani, Daisuke Tanaka, Shunyue Cheng, Hiroshi Toyoda, Kumiko Namba, Tamami Fukushi, Tomoyo Morita, Hisakazu Yanaka (Research Institute of Science and Technology for Society, Japan Science and Technology Agency), Yoichi Sakakihara (Department of Child Care and Education, Ochanomizu University), Kanehisa Morimoto (Graduate School of Medicine, Osaka University), Kayako Nakagawa (Graduate School of Engineering, Osaka University), Shoji Itakura (Graduate School of Letters, Kyoto University), Kiyotaka Tomiwa (Graduate School of Medicine, Kyoto University), Shunya Sogon (The Graduate Division of the Faculty of Human Relations, Kyoto Koka Women’s University), Toyojiro Matsuishi (Department of Pediatrics and Child Health, Kurume University), Tamiko Ogura (Graduate School of Humanities, Kobe University), Masako Okada (Koka City Educational Research Center), Hiroko Ikeda (National Epilepsy Center Shizuoka Institute of Epilepsy and Neurological Disorder), Norihiro Sadato (National Institute for Physiological Sciences, National Institutes of Natural Sciences), Mariko Y. Momoi, Hirosato Shiokawa, Takanori Yamagata (Department of Pediatrics, Jichi Medical University), Tadahiko Maeda, Tohru Ozaki (The Institute of Statistical Mathematics, Research Organization of Information and Systems), Tokie Anme (Graduate School of Comprehensive Human Sciences, University of Tsukuba), Takahiro Hoshino (Graduate School of Arts and Sciences, The University of Tokyo), Osamu Sakura (Interfaculty Initiative in Information Studies, The University of Tokyo), Yukuo Konishi (Department of Infants’ Brain & Cognitive Development, Tokyo Women’s Medical University), Katsutoshi Kobayashi (Center for Education and Society, Tottori University), Tatsuya Koeda, Toshitaka Tamaru, Shinako Terakawa, Ayumi Seki, Ariko Takeuchi (Faculty of Regional Sciences, Tottori University), Hideo Kawaguchi (Advanced Research Laboratory, Hitachi, Ltd.), Sonoko Egami (Hokkaido University of Education), Yoshihiro Komada (Department of Pediatric and Developmental Science, Mie University Graduate School of Medicine Institute of Molecular and Experimental Medicine), Hatsumi Yamamoto, Motoki Bonno, Noriko Yamakawa (Clinical Research Institute, Mie-chuo Medical Center, National Hospital Organization), Masatoshi Kawai (Institute for Education, Mukogawa Women’s University), Yuko Yato (College of Letters, Ritsumeikan University), Koichi Negayama (Graduate School of Human Sciences, Waseda University).
